# Evaluating physicians’ teaching perspectives and demonstration of core competencies in clinical shadowing

**DOI:** 10.1186/s12909-025-08321-1

**Published:** 2025-12-29

**Authors:** Chiao-Ling Tsai, Chun-Ta Huang, Chih-Wei Yang, Yen-Lin Chiu, Chia-Ter Chao, Mong-Wei Lin, Chao-Chi Ho, Huey-Ling Chen, Chiun Hsu

**Affiliations:** 1 Division of Radiation Oncology, Department of Oncology, National Taiwan University Hospital, Taipei, Taiwan; 2 Graduate Institute of Medical Education and Bioethics, National Taiwan University College of Medicine, Taipei, Taiwan; 3 Department of Internal Medicine, National Taiwan University Hospital, No. 7, Chung-Shan South Road, Taipei, Taiwan; 4 Department of Emergency Medicine, National Taiwan University Hospital, Taipei, Taiwan; 5 Department of Medical Education, National Taiwan University Hospital, Taipei, Taiwan; 6 Department of Surgery, National Taiwan University Hospital, Taipei, Taiwan; 7 Department of Pediatrics, National Taiwan University Cancer Center, Taipei, Taiwan; 8 Department of Medical Oncology, National Taiwan University Cancer Center, Taipei, Taiwan

**Keywords:** Clinical shadowing, Core competency, Teaching perspectives inventory, Teaching style, Undergraduate medical education

## Abstract

**Background:**

Clinical shadowing serves as a crucial curriculum for medical students, enhancing their clinical proficiency and fostering professional socialization. This study examined the Teaching Perspective Inventory (TPI) of shadowed physicians and six core competencies they chose to exhibit. Additionally, we dug into the association between the TPI, preferred competencies demonstrated, and physician characteristics.

**Methods:**

At the College of Medicine, National Taiwan University (NTUCM), participation in clinical shadowing is mandatory for second- and third-year medical students. Shadowed physicians volunteered to participate. An online, anonymous questionnaire was distributed to participating physicians to gather information about their age, sex, specialties, experience in clinical shadowing, and TPI. These physicians were also asked about six core competencies they preferred to demonstrate during shadowing sessions.

**Results:**

Out of 203 questionnaires distributed, 134 (66%) were collected and analyzed. Apprenticeship, with a prevalence of 46%, emerged as the most dominant teaching perspective, while Transmission, at 53%, was the most common recessive perspective. The top core competencies that physicians preferred to demonstrate during shadowing activities were Interpersonal and communication skills (70%), Systems-based practice (66%), and Professionalism (60%). Factors such as age, gender, and specialties of physicians had a certain impact on the core competencies they chose to exhibit, such as Interpersonal and communication skills or Systems-based practice.

**Conclusions:**

Physicians predominantly demonstrated the Apprenticeship teaching perspective and favored showcasing Interpersonal and communication skills, Systems-based practice, and Professionalism during preclinical medical student shadowing. Physician characteristics influenced their TPI and core competencies displayed. Overall, our findings contribute to paving the path for future evidence-based educational reform.

**Clinical trial number:**

Not applicable.

**Supplementary Information:**

The online version contains supplementary material available at 10.1186/s12909-025-08321-1.

## Introduction

Clinical shadowing is an experience characterized by observing a licensed healthcare provider as they care for patients [1]. The objective is to provide an insight into the field of medicine, a sneak peek into a particular medical specialty along with its potential daily duties. Additionally, it allows the healthcare provider to demonstrate professional conduct and exceptional patient care [1]. During their preclerkship training, medical students are often obligated to fulfill a certain number of hours in clinical shadowing. The structure of clinical shadowing within medical school programs can range from formal experiences arranged by faculty, which come with compulsory requirements based on hours or specialties, to flexible opportunities organized by students themselves during their free time. Clinical shadowing is perceived by medical students as a valuable tool for career exploration. A plethora of literature underscores its importance in enhancing clinical proficiency, facilitating professional socialization, and fostering mentor relationships [2, 3]. 

The Teaching Perspectives Inventory (TPI) is a tool that assesses teachers’ profiles across five unique teaching perspectives labeled Transmission, Apprenticeship, Developmental, Nurturing, and Social Reform [4]. It prompts teachers to reflect on their teaching beliefs, intentions, and actions [4]. The TPI serves as a valuable resource for fostering self-reflection, crafting teaching philosophy statements, stimulating discussions about teaching methods, and acknowledging diverse forms of teaching excellence [5]. As a complimentary, self-administered, and self-evaluated tool, the TPI encourages a multifaceted comprehension of teaching and provides respondents with a more precise language to contemplate their own teaching practices and those of their peers [6, 7]. Over a decade of collected responses from over 100,000 participants across more than 100 countries has led researchers to determine that the TPI generally displays the psychometric accuracy and consistency that most investigators would deem as “satisfactory” or even “good” [5]. 

The concept of the six core competencies was first described and endorsed by the Accreditation Council for Graduate Medical Education (ACGME) and the American Board of Medical Specialties (ABMS) in 1999 [8, 9]. The competencies, which include Medical knowledge, Interpersonal and communication skills, Systems-based practice, Patient care, Practice-based learning and improvement, and Professionalism, were developed to define the foundational skills that every practicing physician should possess [8, 9]. These competencies also serve to shape and evaluate the education of residents [9]. Acknowledging the necessity to enhance the transition from undergraduate to graduate medical education, the Coalition for Physician Accountability advocated for a core competency framework applicable to all physicians. In 2014, they introduced a unifying framework that encapsulates six fundamental yet broad competency domains across the spectrum of training [10]. To ensure a seamless transition from undergraduate to graduate medical education, a multi-institutional advisory committee, established in 2021, encompassing the Association of American Medical Colleges (AAMC), the American Association of Colleges of Osteopathic Medicine (AACOM), and the ACGME has also pledged to adopt this six core competency framework in undergraduate medical education programs [11]. 

Pedagogical Content Knowledge (PCK), a concept introduced by Lee Shulman in the 1980s [12], refers to the unique integration of subject matter expertise and pedagogical skill that enables teachers to make complex concepts comprehensible to students. It emphasizes the ability of teachers to adapt their teaching approaches to the nature of the content and the learning needs of their students. In essence, PCK represents the intersection between knowing the subject and understanding the most effective ways to teach it. Given the importance of clinical shadowing as an early clinical contact in the educational journey of medical undergraduates [13], it is imperative for physicians involved in shadowing to identify characteristics of their own teaching styles. Only by understanding their own perspectives on teaching can they fully grasp, thoughtfully consider, and incorporate additional styles and approaches where appropriate. In this context, society guidelines have recommended the TPI as the preferred self-assessment tool [14]. As one of the programs encouraged by the ACGME to engage students earlier and farther upstream in the career pathway [15], clinical shadowing should serve as a venue and opportunity for physicians to role model core competencies for medical students. While both the TPI and ACGME core competencies have been widely utilized in medical education research, few studies have examined their intersection within the context of preclinical shadowing, where students primarily learn through observation rather than structured instruction. Accordingly, our study adopted an exploratory approach to address the lack of empirical understanding of how physicians’ teaching perspectives influence the professional competencies they model for medical students during shadowing experiences. The study had two primary aims: first, to identify which competencies physicians being shadowed sought to demonstrate during these sessions; and second, to explore whether physicians with particular teaching perspectives tended to emphasize specific competencies in their teaching practices.

## Methods

### Study settings and participants

This study was undertaken at the College of Medicine, National Taiwan University (NTUCM) in academic year 2022 − 2023. Our institution has been conducting clinical shadowing programs since 2012. Participation in these activities is compulsory for our second- and third-year medical students, who are required to complete a two-hour shadowing session each semester. Before starting the program, students receive a brief introduction to the six core competencies and are then tasked with observing these competencies in practice. The aim of the program is to enable preclinical students to comprehend physicians’ daily responsibilities and to reflect on the essential core competencies they need to acquire. The physicians being shadowed are from hospitals affiliated with NTUCM, and they volunteer to take part in these activities.

### The TPI

Conceived by Pratt and Collins in 2000 [7], the TPI measures respondents’ beliefs, intentions, and actions related to five contrasting views of teaching, identifying both dominant and recessive perspectives. The TPI has undergone thorough examination and application by educators across a wide range of disciplines, establishing its credibility and dependability [5, 16]. It has been featured in numerous scholarly works for its applications in faculty development, teaching evaluations, instructional enhancements, peer teaching reviews, and higher education research [14, 17, 18]. Significantly, the TPI has been instrumental in assessing the clinical teaching viewpoints of medical teachers and clinicians across various specialties [19–21]. 

When engaging in professional development or assessing teaching, it is crucial to consider the attributes of teaching perspectives. Additionally, we must recognize the plurality of good in teaching as a social act with moral and cultural dimensions, shaped by tradition and time [22]. Furthermore, each of these dimensions should be acknowledged through the constructs that constitute the TPI [5]. In this context, we believe that the TPI is the ideal tool to delineate the teaching perspectives of physicians involved in clinical shadowing.

The TPI consists of 45 items evenly distributed across five teaching perspective scales, with each item rated on a 5-point Likert scale [16]. This scoring yields individual perspective scores ranging from 9 to 45 and an overall composite score from 45 to 225. To classify dominant and recessive perspectives, the mean and standard deviation of each participant’s five perspective scores were calculated, with the overall average derived from these scores. Perspectives scoring one standard deviation above the overall average were identified as dominant, whereas those one standard deviation below the average were classified as recessive [16]. This approach enabled individualized assessment of teaching philosophy relative to each participant’s unique profile.

### Survey

Given the goal of recruiting over one hundred physicians from multiple institutions, many of whom have demanding clinical responsibilities, conducting in-person interviews was not feasible. To ensure broad participation and accommodate physicians’ schedules, an online questionnaire was utilized as a practical and efficient data collection method. Moreover, the survey was administered anonymously to encourage honest disclosure and minimize socially desirable responding, thereby enhancing the credibility of the data collected. An online, anonymous questionnaire was then distributed to the physicians being shadowed to gather information about their age, sex, specialties, experience in clinical shadowing, and TPI. In this study, specialties were categorized as medical (Internal Medicine, Family Medicine, Neurology, Pediatrics, Psychiatry, Medical Genetics, Oncology, Physical Medicine and Rehabilitation, Geriatric Medicine), surgical (Surgery, Obstetrics and Gynecology, Ophthalmology, Urology, Dermatology, Anesthesiology, Orthopedic Surgery, Traumatology), and others (Pathology, Nuclear Medicine, Radiology, Laboratory Medicine). These physicians were also asked about the six core competencies they preferred to demonstrate during shadowing sessions (see Additional file 1).

The Traditional Chinese version of the TPI (see Additional file 2) [23], adapted from the Simplified Chinese and English versions, was used in this study [7]. To validate the translated tool, it underwent both face and content validity evaluations by nine specialists in medical education and language translation. These professionals scrutinized the items for their suitability, relevance, and comprehensibility within the context of the local healthcare education system. The items from the TPI were evaluated on a 4-point Likert scale, with 1 being very inappropriate and 4 being very appropriate. The experts’ recommendations were incorporated during the questionnaire revision process. All 45 original items were deemed relevant and retained. The internal structure validity of the TPI was assessed through several metrics. The content validity indices (CVI) were presented at both the item-level (I-CVI) and the scale-level (S-CVI) [24, 25]. The I-CVI of the 45 items ranged from 0.78 to 1, and the S-CVI was 0.98 [23], indicating acceptable content validity for the tool. Furthermore, the internal consistency reliability was high, as demonstrated by the Cronbach’s alpha values ranging from 0.923 to 0.931 across the five perspectives [23]. The TPI evaluates all five teaching perspectives of respondents and pinpoints one (or two) dominant perspectives, as well as recessive perspectives for them [5]. 

### Ethics

The survey, conducted as part of an educational reform initiative, collected only anonymous data. Consequently, the Research Ethics Committee of National Taiwan University Hospital (202401224 W) reviewed the protocol and granted an exemption from full-board review, waiving the requirement for participant consent. All study procedures were carried out in strict adherence to the principles of the Declaration of Helsinki. A part of the study results has been reported elsewhere [23]. 

### Statistical analysis

Data were depicted as proportions and analyzed using the χ^2^ test or Fisher’s Exact test, as appropriate. All statistical evaluations were performed utilizing SPSS Statistics for Windows (Version 22.0; IBM Corp., Armonk, NY, US). A two-tailed P value of less than 0.05 was indicative of statistical significance. Given that the analyses probing the association between the TPI and six core competencies were exploratory in nature, no statistical adjustment was made for multiple comparisons.

## Results

### Characteristics of participants and their TPI

A total of 203 questionnaires were disseminated to the physicians being shadowed, out of which 134 (66%) valid responses were retrieved for analysis. More than half of the participants (*N* = 78, 58%) were within the age range of 40 − 50 years, and a significant majority (*N* = 108, 81%) were male (Table 1). Approximately two-thirds (*N* = 89, 66%) and one-fourth (*N* = 33, 25%) of the shadowed physicians were from medical and surgical specialties, respectively.

**Table 1:** Characteristics of participating physicians

	Total	Medical specialties	Surgical specialties	Other specialties	*P* value
	*N* = 134	*N* = 89	*N* = 33	*N* = 12	
Age, years					
<40	23 (17)	13 (15)	6 (18)	4 (33)	0.635
40 − 50	78 (58)	53 (60)	19 (58)	6 (50)	
>50	33 (25)	23 (26)	8 (24)	2 (17)	
Male sex	108 (81)	68 (76)	30 (91)	10 (83)	0.192
Shadowing sessions					
<3	49 (37)	31 (35)	12 (36)	6 (50)	0.870
3 − 6	46 (34)	31 (35)	11 (33)	4 (33)	
>6	39 (29)	27 (30)	10 (30)	2 (17)	

**Fig. 1:**
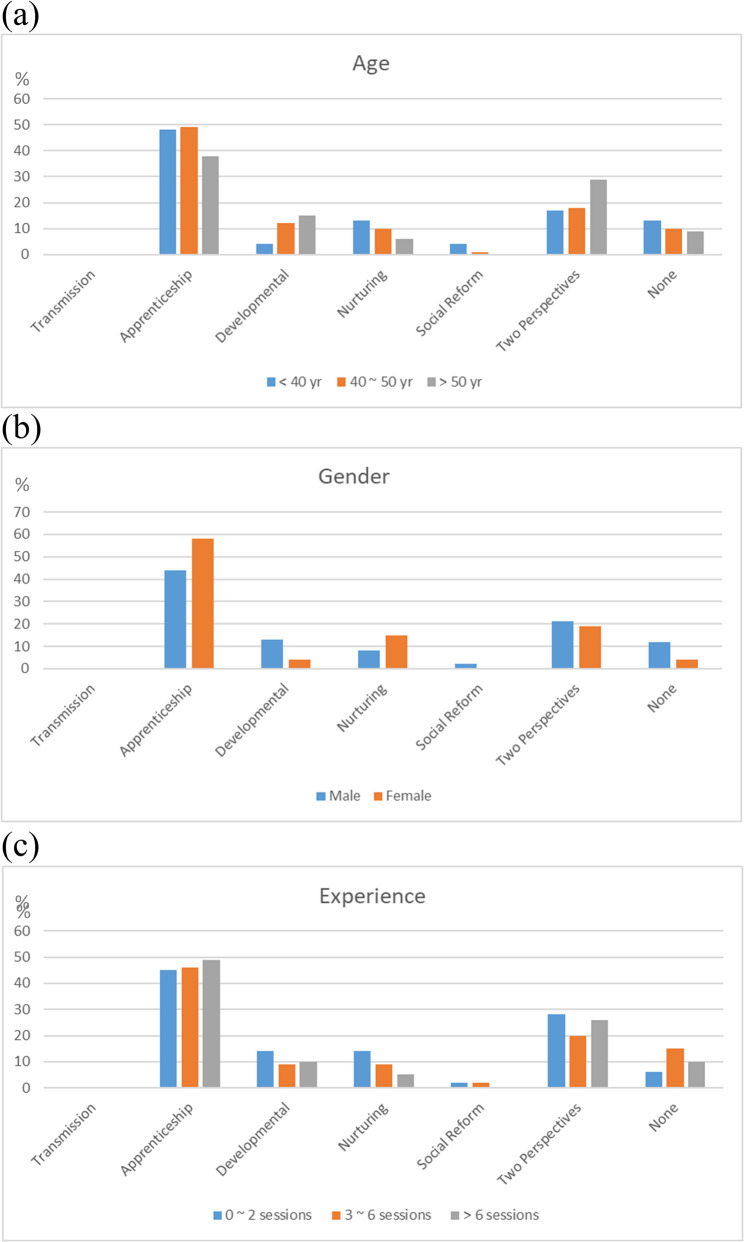
Dominant teaching perspectives in relation to (**a**) age, (**b**) gender, and (**c**) clinical shadowing experience

**Fig. 2:**
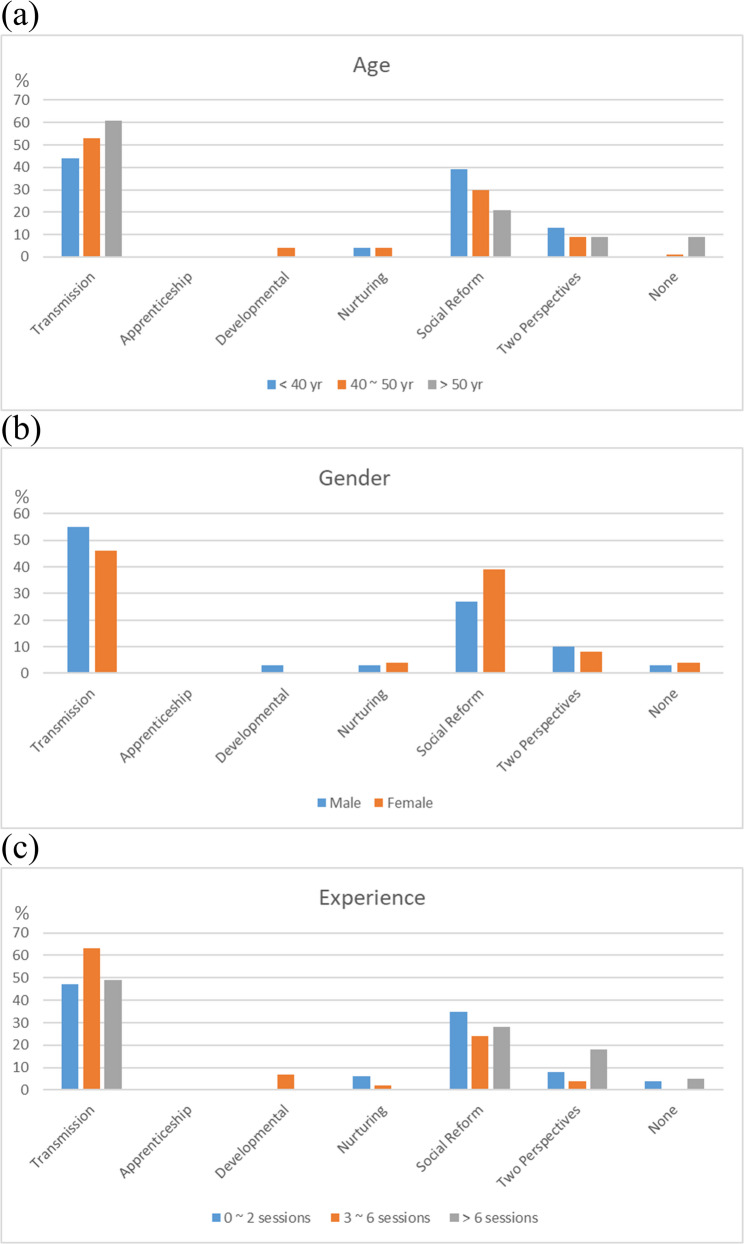
Recessive teaching perspectives in relation to (**a**) age, (**b**) gender, and (**c**) clinical shadowing experience

Overall, Apprenticeship (*N* = 62, 46%) emerged as the most prevalent dominant teaching perspective among the participants, succeeded by those with two dominant perspectives (*N* = 28, 21%) and with Developmental (*N* = 15, 11%). The distribution of dominant teaching perspectives was consistent across specialties (Table 2), age, gender, and clinical shadowing experience (Fig. 1a − c). Regarding recessive teaching perspectives, a comparable distribution was also noted across diverse age categories, genders, and levels of clinical shadowing experience (Fig. 2a − c), with Transmission (*N* = 71, 53%) and Social Reform (*N* = 39, 29%) the most prevailing ones (Table 2). Notably, physicians from other specialties were more likely to identify Social Reform as a recessive teaching perspective compared to those from Surgical specialties (58% vs. 18%; post-hoc *P* = 0.009).

**Table 2:** Teaching perspectives inventory of participating physicians

	Total	Medical specialties	Surgical specialties	Other specialties	*P* value
	*N* = 134	*N* = 89	*N* = 33	*N* = 12	
Dominant perspectives					
Transmission	0 (0)	0 (0)	0 (0)	0 (0)	NA
Apprenticeship	62 (46)	40 (45)	14 (42)	8 (67)	0.322
Developmental	15 (11)	8 (9)	6 (18)	1 (8)	0.337
Nurturing	13 (10)	9 (10)	3 (9)	1 (8)	0.999
Social Reform	2 (2)	2 (2)	0 (0)	0 (0)	0.999
Two perspectives	28 (21)	20 (23)	6 (18)	2 (17)	0.814
None	14 (10)	10 (11)	4 (12)	0 (0)	0.677
Recessive perspectives					
Transmission	71 (53)	44 (49)	22 (67)	5 (42)	0.170
Apprenticeship	0 (0)	0 (0)	0 (0)	0 (0)	NA
Developmental	3 (2)	3 (3)	0 (0)	0 (0)	0.670
Nurturing	4 (3)	4 (5)	0 (0)	0 (0)	0.708
Social Reform	39 (29)	26 (29)	6 (18)	7 (58)	0.039
Two perspectives	13 (10)	8 (9)	5 (15)	0 (0)	0.324
None	4 (3)	4 (5)	0 (0)	0 (0)	0.708

*NA* Not applicable

### Demonstration of six core competencies

Interpersonal and communication skills (70%), Systems-based practice (66%), and Professionalism (60%) emerged as the top core competencies that physicians being shadowed chose to demonstrate during shadowing activities (Table 3). Intriguingly, a significantly larger proportion of physicians from medical specialties (82%) expressed a preference for showcasing Interpersonal and communication skills compared to their counterparts from surgical (49%) and other specialties (42%; post-hoc *P* < 0.001 and 0.002, respectively, vs. medical specialties). Conversely, participants from surgical specialties showed a stronger inclination towards exhibiting Systems-based practice than those from medical specialties (85% vs. 58%; *P* = 0.006).

**Table 3:** Six core competencies demonstrated by participating physicians

	Total	Medical specialties	Surgical specialties	Other specialties	*P* value
	*N* = 134	*N* = 89	*N* = 33	*N* = 12	
Medical knowledge	20 (15)	14 (16)	5 (15)	1 (8)	0.932
Interpersonal and communication skills	94 (70)	73 (82)	16 (49)	5 (42)	< 0.001
Systems-based practice	88 (66)	52 (58)	28 (85)	8 (67)	0.024
Patient care	38 (28)	25 (28)	12 (36)	1 (8)	0.182
Practice-based learning and improvement	49 (37)	30 (34)	14 (42)	5 (42)	0.626
Professionalism	81 (60)	56 (63)	18 (55)	7 (58)	0.694

**Fig. 3:**
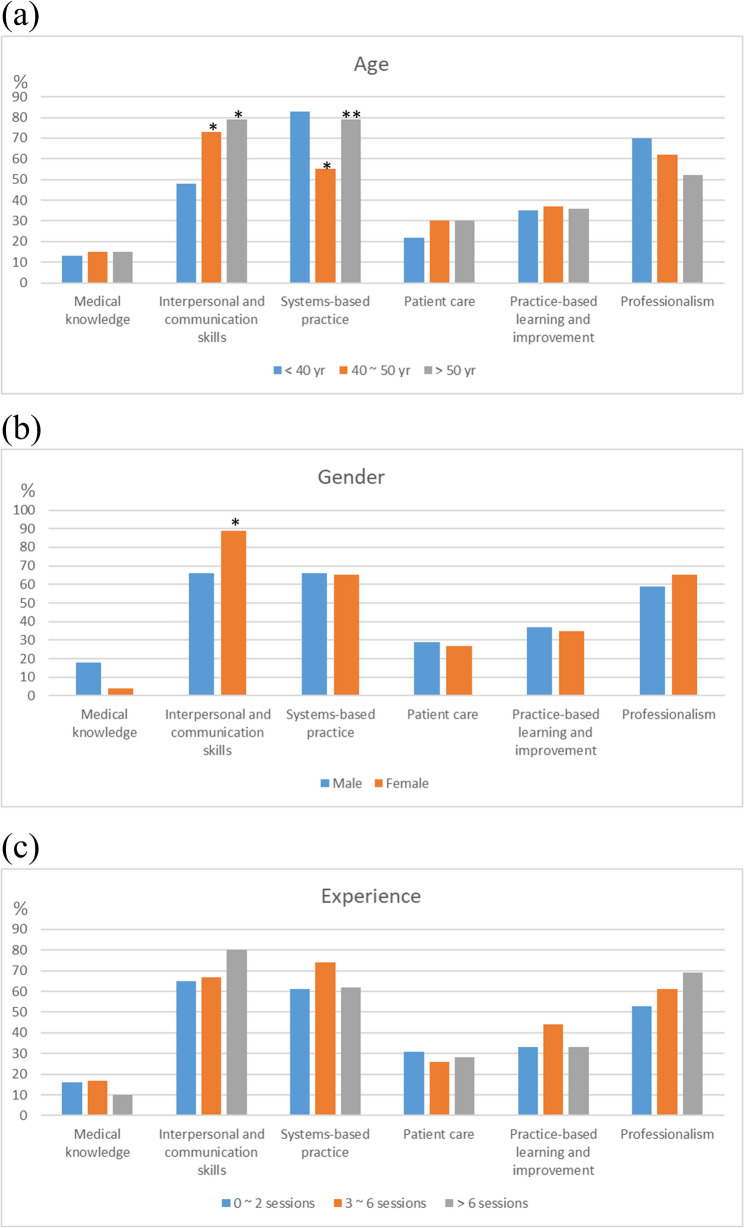
Demonstration of six core competencies in relation to (**a**) age, (**b**) gender, and (**c**) clinical shadowing experience ^*^P < 0.05 compared to the first group; ^**^P < 0.05 compared to the second group

With respect to age (Fig. 3a), physicians aged 40 − 50 (73%) and above 50 years (79%) were more inclined to demonstrate Interpersonal and communication skills than those under 40 years of age (48%). Systems-based practice was more frequently demonstrated by participants aged under 40 (83%) and over 50 years (79%) than those aged 40 − 50 years (55%). In terms of gender, female physicians (89%) showed a greater preference for demonstrating Interpersonal and communication skills compared to their male counterparts (66%; Fig. 3b). Clinical shadowing experience did not influence the competencies that physicians chose to demonstrate (Fig. 3c).

### Association between TPI and demonstrated competencies

Table 4 illustrates the association between the participants’ dominant teaching perspectives and the competencies they prefer to demonstrate. The singular association identified was that physicians being shadowed, who had no dominant teaching perspective, were more inclined to exhibit Medical knowledge compared to those with other perspectives (*P* = 0.037). Conversely, Table 5 presents the association between the physicians’ recessive teaching perspectives and the competencies they prefer to demonstrate. It was exclusively observed that physicians with a recessive teaching perspective of Transmission were less likely to demonstrate Medical knowledge compared to those with other perspectives (*P* = 0.001).

**Table 4:** Association between dominant teaching perspectives and six core competencies

	Transmission	Apprenticeship	Developmental	Nurturing	Social Reform	Two perspectives	None
Six core competencies	(*N* = 0)	(*N* = 62)	(*N* = 15)	(*N* = 13)	(*N* = 2)	(*N* = 28)	(*N* = 14)
Medical knowledge	0 (0)	7 (11)	2 (13)	2 (15)	0 (0)	4 (14)	5 (36)^*^
Interpersonal and communication skills	0 (0)	43 (69)	12 (80)	9 (69)	2 (100)	20 (71)	8 (57)
Systems-based practice	0 (0)	45 (73)	10 (67)	7 (54)	0 (0)	19 (68)	7 (50)
Patient care	0 (0)	14 (23)	5 (33)	3 (23)	1 (50)	9 (32)	6 (43)
Practice-based learning and improvement	0 (0)	24 (39)	5 (33)	6 (46)	1 (50)	8 (29)	5 (36)
Professionalism	0 (0)	36 (58)	10 (67)	6 (46)	2 (100)	20 (71)	7 (50)

^*^P < 0.05

**Table 5:** Association between recessive teaching perspectives and six core competencies

	Transmission	Apprenticeship	Developmental	Nurturing	Social Reform	Two perspectives	None
Six core competencies	(*N* = 71)	(*N* = 0)	(*N* = 3)	(*N* = 4)	(*N* = 39)	(*N* = 13)	(*N* = 4)
Medical knowledge	4 (6)^*^	0 (0)	2 (67)	0 (0)	9 (23)	3 (23)	2 (50)
Interpersonal and communication skills	48 (68)	0 (0)	2 (67)	3 (75)	28 (72)	10 (77)	3 (75)
Systems-based practice	45 (63)	0 (0)	2 (67)	2 (50)	27 (69)	10 (77)	2 (50)
Patient care	23 (32)	0 (0)	1 (33)	1 (25)	8 (21)	4 (31)	1 (25)
Practice-based learning and improvement	24 (34)	0 (0)	2 (67)	1 (25)	16 (41)	5 (39)	1 (25)
Professionalism	45 (63)	0 (0)	2 (67)	3 (75)	19 (49)	10 (77)	2 (50)

^*^P < 0.05

## Discussion

The TPI guides the process of critical reflection by establishing a foundational layer of information. It also articulates the educators’ personal beliefs about learning, knowledge, and the societal role embodied by a teacher [16]. For the first time, we delved into the TPI of physicians involved in the shadowing activities of second- and third-year medical students. Apprenticeship emerged as the primary dominant teaching perspective among participants, while Transmission was the most common recessive teaching perspective, followed by Social Reform. Apprenticeship, with its deep roots in medicine, continues to play a pivotal role in medical education [26, 27]. Furthermore, NHS England recently introduced a “medical doctor degree apprenticeship”, providing an alternative pathway into medicine as a career, beyond the traditional medical degree route [28]. During shadowing activities, observers acquire insights by directly witnessing the realities of their prospective career, encompassing but not limited to, patient-physician interactions, procedures, and examinations [29]. Given this context, it is understandable that Apprenticeship emerged as the dominant teaching perspective among our participants. The concept of Transmission encapsulates the notion that effective teaching necessitates mastery of the subject matter and the ability to convey information in a structured and organized manner [16]. As medical students participating in physical shadowing are not expected to acquire any specific body of knowledge, it was logical to observe Transmission as the most prevalent recessive teaching perspective among the shadowed physicians.

The dual role of clinical physicians as both healthcare providers and educators is acknowledged, which may lead to stress and impact their identity as medical teachers [30]. Although physicians are usually well-prepared for their clinical roles, many lack knowledge of educational principles and teaching strategies, leaving them inadequately prepared for this additional professional role [31]. The AMEE guide has been published to address the challenges of teaching in the clinical environment [32]. It suggests that physicians involved in clinical teaching should have some knowledge of different learning styles and adapt their teaching methods to suit various students [32]. In this context, identifying one’s own TPI is crucial for understanding one’s teaching techniques and increasing awareness of strengths and weaknesses. There is no straightforward formula for matching teaching approaches with students’ learning preferences, but when educators strive to diversify their teaching techniques to cater to different learning styles, it can foster a better connection with students [33–35]. This, in turn, can make students more comfortable and eager to learn [33–35]. Take experiential learning as an instance: it is generally better for physicians to adopt an Apprenticeship approach rather than a Transmission approach. Yet, it remains somewhat ambiguous whether this actually enhances students’ academic outcomes [36, 37]. In terms of clinical shadowing, one of the modalities for experiential learning, we found a similar pattern of teaching styles—Apprenticeship as the dominant and Transmission as the most prevalent recessive—suggesting our physicians may have adapted their teaching styles to the teaching scenarios [38]. Given the need to adapt teaching styles across different academic disciplines, each perspective is best suited to specific educators and the contexts in which they teach [38]. While the ACGME has recognized the significance of six core competencies across all levels of medical education and published the Milestones Guidebook to promote Competency-Based Medical Education, the methods of teaching, evaluating, modeling, and enforcing these competencies are left to the discretion of program administrators within their respective programs [8, 11, 15]. Clinical shadowing, which is not theoretically designed to deliver any formal curriculum to observers, can serve as a crucial platform for imparting the hidden curriculum [39]. In our institution, medical students are specifically instructed to observe these six core competencies. Therefore, it is essential to understand which core competencies physicians choose to exhibit during shadowing activities. Approximately two-thirds of the participating physicians in the present study reported Interpersonal and communication skills, Systems-based practice, and Professionalism as the competencies they prefer to demonstrate. These findings align with the nature of a shadowing course for preclinical medical students, which is essentially an observership [40]. 

Fostering professionalism among preclinical medical students poses challenges due to the complexity of the concept and its contextual application. Additionally, the multifaceted nature of professionalism complicates its teaching and assessment in undergraduate medical education. Despite efforts, there is no universally accepted theoretical framework or standardized teaching and assessment tools for professionalism in medical education [41, 42]. Professionalism serves as a fundamental competency for undergraduate medical students on a global scale [43]. Both teachers and students identify role modeling as the primary approach for cultivating professionalism [44–46]. While classroom faculty and peers can serve as role models for preclinical medical students, modeling by physicians in patient-care settings offers an excellent opportunity to instill professionalism [44]. Therefore, it is particularly gratifying and educationally significant for our study to find that a majority of physicians are eager to demonstrate Professionalism during shadowing. This program could potentially serve as an early, if not initial, platform for medical students to observe and assimilate professional behavior and conduct in authentic clinical environments.

In relation to Systems-based practice, students are expected to demonstrate an understanding of and responsiveness to the broader context and structure of healthcare. This includes recognizing the social and structural determinants of health and effectively utilizing additional resources to deliver optimal healthcare [15]. Yet, unlike the considerable advancements made in other ACGME competencies, Systems-based practice, even 20 years after its establishment, persists as a crucial concept lacking significant traction, and struggles to gain equal recognition among both teachers and students [47, 48]. From the perspective of medical students, they may be less inclined to endorse the inclusion of this topic, often perceiving it as less relevant to licensing exams or success in residency matching, or as providing fewer benefits for their future careers [49]. In this study, a notable number of physicians opted to demonstrate Systems-based Practice during their shadowing sessions, providing preclinical medical students with the chance to appreciate and contemplate the significance of Systems-based Practice in undergraduate medical education.

Our study further reveals that physicians with medical specialties tended to showcase Interpersonal and communication skills, while those with surgical specialties were more inclined to demonstrate Systems-based practice. This difference may reflect the inherent variations in physician duties and shadowing settings between the two categories of specialties. For example, medical specialties were more likely to demonstrate Interpersonal and communication skills during shadowing at the outpatient clinics, whereas surgical specialties tended to display Systems-based practice in operating theater activities. Interestingly, the age and gender of physicians also impacted the core competencies they chose to exhibit—namely, Interpersonal and communication skills and Systems-based practice—although the reasons behind these discrepancies remain to be determined. Additionally, the teaching perspectives of physicians, either dominant or recessive, may influence the core competency of Medical knowledge that they preferred to convey or downplay during shadowing sessions. In summary, these findings support evidence-based educational reform as we incorporate clinical shadowing into the curriculum, albeit as a hidden component [50, 51]. 

There are several limitations to this study that warrant discussion. First, participation in the online survey was voluntary, not mandatory. As a result, the findings of the survey may be subject to non-response bias [52]. However, our response rate of 66% is deemed acceptable for research purposes and should not compromise the precision of our measurements [53, 54]. Second, due to the limited number of participants from diverse backgrounds, our study might not have sufficient power to detect the complex relationships among physicians’ characteristics, their TPI, and the core competencies they prefer to demonstrate. However, as a pioneering study in the field of clinical shadowing, our work could inspire more educational researchers to delve into similar studies with the aim of enhancing and refining medical education. In perspective, a formative quantitative assessment of students’ experiences with the six core competencies during clinical shadowing has been underway. We will present updated data in the future. Third, our shadowing program consists of a single two-hour session per student across multiple clinical disciplines within one academic semester. As such, the findings primarily describe local experiences, and their transferability to other contexts or institutions may be limited. Fourth, the outcome variable “preferred competency demonstration” was based solely on self-reported data. As with all questionnaire-based studies, this introduces potential bias, including the influence of socially desirable responses. While strategies such as ensuring anonymity and maintaining confidentiality were implemented to reduce this bias, the lack of observational or triangulated data limits the robustness of our findings. Finally, given the cross-sectional and self-reported nature of the data, all identified associations should be interpreted as exploratory and non-causal. The study was designed to generate hypotheses and provide descriptive insights rather than to establish generalizable conclusions or causal relationships.

## Conclusions

In summary, this study reveals that Apprenticeship was the predominantly dominant teaching perspective while Transmission was the major recessive perspective for participating physicians during the shadowing activities of preclinical medical students. We also discovered that Interpersonal and communication skills, Systems-based practice, and Professionalism were the core competencies that physicians preferred to demonstrate to the observing students. Several physician characteristics were found to be relevant to their TPI and the core competencies they chose to exhibit. Furthermore, a specific teaching perspective, whether dominant or recessive, was correlated with certain competencies that were either displayed or not displayed. Collectively, our study findings assist physicians and program directors in understanding teachers’ TPI and the core competencies they prefer to demonstrate towards physical shadowing, thereby paving the way for future evidence-based educational reform.

## Supplementary Information

Additional file 1. Questionnaire (English Version). Description of data: A questionnaire assessing participant demographics, professional background, and ACGME core competency teaching priorities.

Additional file 2. Traditional Chinese version of the Teaching Perspectives Inventory (TPI). Description of data: Traditional Chinese version of the Teaching Perspectives Inventory (TPI).

## Data Availability

The datasets used and/or analysed during the current study are available from the corresponding author on reasonable request.

## Abbreviations

AACOM: American Association of Colleges of Osteopathic Medicine
AAMC: American Board of Medical Specialties
ABMS: American Board of Medical Specialties
ACGME: Accreditation Council for Graduate Medical Education
CVI: Content validity indices
I-CVI: Item-level content validity indices
NTUCM: College of Medicine, National Taiwan University
PCK: Pedagogical Content Knowledge
S-CVI: Scale-level content validity indices
TPI: Teaching Perspective Inventory
