# Supplementary material for: Evaluating physicians’ teaching perspectives and demonstration of core competencies in clinical shadowing
Source: BMC Med Educ. 2025 Dec 29;25:1718. doi: 10.1186/s12909-025-08321-1 (PMC12751530; doi:10.1186/s12909-025-08321-1)
Supplement: Supplementary file 1 — Additional file 1. Questionnaire (English Version). Description of data: A questionnaire assessing participant demographics, professional background, and ACGME core competency teaching priorities. [file 12909_2025_8321_MOESM1_ESM.docx]

**Questionnaire (English Version)**

**Please check the box next to the statement that best fits you.*

**PART I: Basic and Background Information**

Sex:

□Male

□Female

Age:

□< 40 years old

□40 to 50 years old

□> 50 years old

Department/Specialty:

□Medical

□Surgical

□Others (Laboratory Medicine, Emergency Department, etc.)

What is your prior experience supervising 2nd or 3rd-year medical students during clinical shadowing?

□< 3 sessions

□3-6 sessions

□7 sessions or more

**PART II:**

Regarding your clinical shadowing experience with 2nd and 3rd-year medical students, which aspect(s) of the ACGME Six Core Competencies do you most wish to convey (transmit or teach) to the medical students?

□Medical Knowledge

□Interpersonal and Communication Skills

□Systems-Based Practice

□Patient Care

□Practice-Based Learning and Improvement

□Professionalism
