# Supplementary material for: Evaluating physicians’ teaching perspectives and demonstration of core competencies in clinical shadowing
Source: BMC Med Educ. 2025 Dec 29;25:1718. doi: 10.1186/s12909-025-08321-1 (PMC12751530; doi:10.1186/s12909-025-08321-1)
Supplement: Supplementary file 2 — Additional file 2. Traditional Chinese version of the Teaching Perspectives Inventory (TPI). Description of data: Traditional Chinese version of the Teaching Perspectives Inventory (TPI). [file 12909_2025_8321_MOESM2_ESM.docx]

**Additional file 1. Traditional Chinese version of the Teaching Perspectives Inventory (TPI)**

| 從以下觀點中選出一個您認為最有代表性的說法，將相對應的字母填入方括弧中。 | |  |  |  |  |
| --- | --- | --- | --- | --- | --- |
| 不同的教育信念（你相信教課或教學是什麼？ ） | 同意程度 | | | | |
| 請從以下觀點中選出一個您認為最有代表性的說法。 | 完全不同意 | 不太同意 | 普通 | 同意 | 非常同意 |
| 1. 只有先定預期目標，學習才會有動力。 |  |  |  |  |  |
| 2. 要成為一名有效的教學者， 必須是一位有效的實務工作者。 |  |  |  |  |  |
| 3. 最重要的是，學習要建立在現有知識之上。 |  |  |  |  |  |
| 4. 認可學習者的情緒反應很重要。 |  |  |  |  |  |
| 5. 我把教學的重點放在整個社會的變化上，而不是個別學習者身上。 |  |  |  |  |  |
| 6. 教師應該是自己所教所教專業的大師級人物。 |  |  |  |  |  |
| 7. 和優秀的實務工作者一起工作是一個最佳的學習過程。 |  |  |  |  |  |
| 8. 教學的中心任務應該是促進學習者思維發生質變。 |  |  |  |  |  |
| 9. 在教學中，我以幫助學習者建立自信心為首要任務。 |  |  |  |  |  |
| 10. 沒有引起社會變化的個人學習是不夠的。 |  |  |  |  |  |
| 11. 教學成效好的教師必須首先是自己所教領域的權威。 |  |  |  |  |  |
| 12. 知識及其應用密不可分。 |  |  |  |  |  |
| 13. 教學應該建立在人們現有知識之上。 |  |  |  |  |  |
| 14. 在學習過程中，人們所付出的努力也應該像取得好成績一樣受到嘉獎。 |  |  |  |  |  |
| 15. 對我來說，教學不僅僅是一種知識活動，它 同時也是一種道德行為。 |  |  |  |  |  |

|  |  |  |  |  |  |
| --- | --- | --- | --- | --- | --- |
| 不同的教學意圖 （您在教學過程中想努力實現什麼？ ) | 同意程度 | | | | |
| 您怎麼努力完成您的教學或教學？ 對於每個語句，選擇最能代表多久它代表了您的教育意圖的回應。 | 不曾 | 很少 | 有時 | 通常 | 總是 |
| 16.我的目的是幫助學生應考。 |  |  |  |  |  |
| 17. 我的目的是展示在現實環境中怎麼操作或者怎麼工作。 |  |  |  |  |  |
| 18. 我的目的是幫助學習者更全面、多角度，深層次地發展他們的推理方式。 |  |  |  |  |  |
| 19. 我的目的是幫助學習者樹立自信心和自尊心。 |  |  |  |  |  |
| 20. 我的目的是鞭策學習者，讓他們重新認真考慮自己的價值觀。 |  |  |  |  |  |
| 21. 我期望學習者能夠熟練掌握很多與科目相關的知識。 |  |  |  |  |  |
| 22. 我期望學習者能夠知道如何在實際環境中運用所學的知識。 |  |  |  |  |  |
| 23. 我期望學習者能針對學習主題發展新的推理方式。 |  |  |  |  |  |
| 24. 我期望學習者能夠通過我的教學增強自尊心。 |  |  |  |  |  |
| 25. 我期望學習者能夠投身到改革社會的事業。 |  |  |  |  |  |
| 26. 我期望學習者通過我的教學能在考試中取得好成績。 |  |  |  |  |  |
| 27. 我想讓學習者了解真實世界工作的實際情況。 |  |  |  |  |  |
| 28. 我想讓學習者明白事情真的是如何錯綜複雜而又彼此相關。 |  |  |  |  |  |
| 29. 我想在教學中體現一種平衡關係，既對學習者表示關心，又給他們增加學習難度。 |  |  |  |  |  |
| 30. 對於那些人們認為社會上理所當然存在的事情，我想使這些事情變得顯而易見。 |  |  |  |  |  |

|  |  |  |  |  |  |
| --- | --- | --- | --- | --- | --- |
| 不同的教學行為 （您在教學中做什麼？ ) | 同意程度 | | | | |
| 就以下觀點，選擇一個最能代表您經常採用的教學行為。 | 不曾 | 很少 | 有時 | 通常 | 總是 |
| 31. 我在規定時間內準確完成必講內容。 |  |  |  |  |  |
| 32. 我把學科知識與實際環境中的操練或應用聯繫起來。 |  |  |  |  |  |
| 33. 我在教學中提出很多問題。 |  |  |  |  |  |
| 34. 我會在每位參與者的工作或貢獻中找出值得表揚的地方。 |  |  |  |  |  |
| 35. 我利用教學主題向他們傳授更高的理想。 |  |  |  |  |  |
| 36. 我的教學受到課程目標的制約。 |  |  |  |  |  |
| 37. 我代表具有優良執業方法和技能的典範。 |  |  |  |  |  |
| 38. 我會對教學主題的常規理解方式提出挑戰。 |  |  |  |  |  |
| 39. 我鼓勵大家表達他們的情緒與感受。 |  |  |  |  |  |
| 40. 在教學中我更強調價值觀，而不是知識本身。 |  |  |  |  |  |
| 41. 我非常明確地解釋要學些什麼。 |  |  |  |  |  |
| 42. 我設法使新手能跟有經驗的人學習。 |  |  |  |  |  |
| 43. 我鼓勵學習者對彼此的想法提出異議。 |  |  |  |  |  |
| 44. 我跟學生分享我的感受，並期望學生也能這樣做。 |  |  |  |  |  |
| 45. 我會將社會需要改革之處與教學目標聯結。 |  |  |  |  |  |
